# United States Veterinary Medical Association

**Published:** 1892-03

**Authors:** W. Horace Hoskins

**Affiliations:** Secretary


### SOCIETY PROCEEDINGS.

### UNITED STATES VETERINARY MEDICAL ASSOCIATION.

A special meeting of the Comitia Minora of the United States Veterinary Medical Association was held on Saturday evening, February 20th, at the "Arena," 41 West Thirty-first street, New York city.

President Huidekoper called the meeting to order, and the following members answered to their names: Drs. Huidekoper, Robertson, Hoskins, Liautard, Winchester, R. A. McLean, T. B. Rayner, and Wm. Dougherty. Absent—Drs. Williams, Stickney, and Schwartzkopff. To fill vacancies by appointment of the chair—Drs. Miller and Clement. Communications were received from Drs. R. A. McLean, H. B. Adair, and L. McLean, and were laid over until September.

The place of meeting for 1892 was considered, and communications were read, from Dr. Williams favoring Columbus or Pittsburg; from Dr. L. McLean favoring Boston; from Dr. Schwartzkopff favoring Philadelphia; from Dr. Stickney pleading for Boston; and from Drs. Hinkley, Wende, and Blank, advocating Buffalo as a suitable place. Dr. Thos. B. Rayner offered a motion that we meet in Buffalo, seconded by Dr. W. B. E. Miller. It being open for discussion, Dr. Winchester took the floor and strongly pleaded for Boston, giving the warmest invitation on behalf of the Eastern veterinarians, and representing that Massachusetts Veterinary Medical Association presented their unanimous appeal. Dr. Clement followed, advocating Buffalo; followed by Dr. Hoskins, who favored the latter place as the one he thought best would serve the Association's interests to the greatest advantage. Dr. Miller advocated Buffalo as the most politic place for us to meet in anticipation of the meeting in Chicago in 1893. Dr. Dougherty favored Boston, likewise Drs. Huidekoper, R. A. McLean and Liautard. Question on the motion was called for and a rising vote showed four for Buffalo, five against. A motion was then offered by Winchester that we meet in Boston, seconded by Miller, and carried unanimously.

The Secretary offered a motion that we convene in 1892 for three or more days, which, after some discussion, was adopted.

A motion was adopted that a Committee of three be appointed to act in conjunction with the local Committee of Boston to complete the necessary arrangements.

The question of the Association having a display at Chicago was then considered, and after a general expression of opinion it was deemed best that we should not, as an Association, exhibit.

The International meeting of 1893 was then considered, and a full expression of opinion was given as to the best plans to complete the preliminary work, which ended in the adoption of a motion that a committee of nine be appointed with power to act, the President as chairman, to prepare and issue invitations to foreign veterinary schools and societies to send delegates, and to arrange for their local reception and entertainment. Power was given them to make such expenditures as they deemed necessary on behalf of the Association.

A special report was received from the chairman of the Army Legislative Committee, reporting the present bill before Congress, and stating the disposition of the Committee to advocate the passage of this bill as an entering wedge. A motion was adopted that we indorse the action of our Committee on this subject.

Dr. Clement brought up the subject of having all essays and papers to be read before the Association handed in at least one month before the meeting, so that members might be appointed to discuss the same. It was unfavorably received.

On motion the meeting then adjourned.

W. HOKACE HOSKINS, *Secretary*.

